# Impact of intergenerational support on older adults’ care expectations in rural areas in China

**DOI:** 10.3389/fpubh.2024.1423173

**Published:** 2024-11-07

**Authors:** Lianxia Wu, Tong Xie, Weihua Guan, Wei Li

**Affiliations:** ^1^Population Research Institute in School of Social Development, East China Normal University, Shanghai, China; ^2^School of Geography, Nanjing Normal University, Nanjing, China; ^3^Collaborative Innovation Center for Development and Utilization of Geographic Information Resources in Jiangsu Province, Nanjing, China

**Keywords:** intergenerational support, older adults’ care expectations, binomial logit regression, moderation effect, in-depth interview

## Abstract

**Introduction:**

As rural-to-urban migration accelerates, rural areas are experiencing a significant increase in empty-nesters among the older adults. Influenced by traditional concepts of filial piety, older adults in rural areas heavily rely on the family-based old care model, creating a complex interplay between supply and demand for older adults’ care. This study investigates the relationship between intergenerational support and older adults’ care expectations in rural areas. We construct measures of financial and care expectations of older adults’ care to reflect older adults’ intentions and choices.

**Methods:**

This study uses data from the 2018 China Health and Retirement Longitudinal Study and rural case studies to employ a binomial logit regression model, moderation effect analysis, and in-depth interviews.

**Results:**

Our research reveals that the level of financial expectations for older adults’ care is inversely related to the extent of financial support from the parental generation but positively related to the level of financial support from the offspring. However, the older adults’ care expectations do not appear to be influenced by intergenerational support. Furthermore, population characteristics of the parental generation, such as educational attainment, age, and marital status, moderate the relationship between intergenerational support and older adults’ care expectations. Educational attainment negatively moderates the impact of parental financial support on financial expectations, age positively moderates offspring’s financial support on financial expectations. However, age negatively moderates offspring’s financial support on care expectations, while marital status positively moderates offspring’s financial support on care expectations.

**Discussion:**

These findings help to elucidate the older adults’ care expectations of rural residents during the process of urbanization and social transformation, offering family-centered solutions such as ‘promoting cohabitation or proximity of children to their parents, developing diverse older adults’ care models based on different family situations, and ensuring the provision of basic older adults’ care services’ to address the current challenges of rural older adults’ care in China.

## 1 Introduction

The challenge of “supply falling short of demand” (1) in older adults’ care in rural China has become an urgent issue in addressing the challenges of population aging. Between 2000 and 2020, the global proportion of people aged 60 and over generally increased, with China’s aging rate and extent being particularly notable, surpassing that of other developing countries. Research shows that during this period, the proportion of China’s older adults rose from 10.0 to 18.7%, while in other developing countries, it increased from 6.8 to 9.2% (2). In the context of the urban–rural dual system, due to the comparative economic advantages of cities, there has been a continuous outflow of surplus labor from rural areas, with the majority of those moving to cities being young and middle-aged individuals (3, 4). This has led to a significant increase in the number of empty-nest older adults in rural areas, along with a growing demand for older adults’ care. However, the proportion of adult children living with their parents in rural China is less than 40% (5), which is significantly lower than the co-residence rates of over 70% in other developing countries such as the Philippines, Thailand, and Vietnam (6). This reduces the opportunities for the older adults to receive intergenerational support, including daily care and emotional comfort (7). The issues of low older adults’ care standards, uneven distribution of older adults’ care resources, and insufficient development of older adults’ care institutions are also prominent in rural China (8). By the end of 2019, the coverage of registered older adults’ care institutions, community-based older adults’ care facilities, and mutual aid older adults’ care facilities in rural China was only 6.40, 12, and 19%, respectively (9), indicating an urgent need to enhance the capacity of older adults’ care services.

Currently, the three main forms of older adults’ care in China are family-based care, social care, and self-care (10), which coexist and all depend on intergenerational support from children. Influenced by local customs or traditions, the predominant choice in rural areas is family-based care, relying on children. Chinese intergenerational relationships are characterized by a reciprocal “nurturing-support” pattern within parent–child interactions (11), encompassing mutual financial, care, and emotional support. These intergenerational relationships are essentially functional relationships based on parent–child bonds, featuring bidirectional interaction and mutual fulfillment. The rapid development of modernization and urbanization has led to the downsizing of core family structures, which weaken family-based caregiving functions. However, research indicates that family-based caregiving remains predominant. Most parents plan to rely on their children to address future older adults’ care needs, believing that children have moral and legal obligations to provide support (12).

Some scholars define older adults’ care expectations as individuals’ anticipations and prospects regarding their living conditions in old age, based on information collected and analyzed regarding existing older adults’ care systems and current older adults’ care conditions (13). In this paper, older adults’ care expectations primarily pertain to the subjective assumptions of the parental generation concerning financial resource provision and caregiving responsibilities in the future, that is, financial and care expectations of older adults’ care, based on their current intergenerational relationships and living conditions. Within this context, intergenerational support predominantly includes the financial support provided by the younger generation and the financial and caregiving support from the parent generation. With the decline of traditional patriarchal and filial piety culture, the children’s voices in intergenerational caregiving are gradually increasing, and their perspectives are potentially shifting. Will this change parental generation’s older adults’ care expectations and behavioral choices? Will distancing in intergenerational relationships or reduced financial support lower older adults’ expectations of their children’s caregiving in rural areas? How will intergenerational support influence older adults’ caregiving plans and lifestyles in rural areas? This study aims to elucidate how intergenerational support influences older adults’ care expectations and parental generation’s caregiving plans and behavioral choices in rural areas, utilizing the 2018 China Health and Retirement Longitudinal Study (CHARLS) data and case interview information.

## 2 Policy context and literature review

### 2.1 Policy context

As China transitions into an aging society, the 20th CPC National Congress proposed the national strategy of Actively Tackle Population Aging, advocating the development of older adults’ care services and industries to ensure that all older adults have access to basic older adults’ care services, which can provide specific policy background for rural older adults’ care expectations. In 2024, China’s Ministry of Civil Affairs issued the “Guiding Opinions on Accelerating the Development of Rural Older Adults’ Care Services,” emphasizing that the development of rural older adults’ care services is crucial for the happiness of hundreds of millions of rural older adults and is essential for the successful implementation of the national strategy to actively address population aging and the rural revitalization strategy. The guidelines stress the importance of “family responsibility” in this context. With the rapid aging of the population, the rising proportion of older adults, and the increasing prevalence of age-related disabilities and cognitive decline, the demand for service provision to China’s older adults has become more pronounced. There is an urgent need to delve into the older adults’ care service policies in response to population aging (14). Examining the relationship between intergenerational support and filial expectations in rural areas is instrumental in clarifying older adults’ care needs and the developmental direction of rural older adults amidst urbanization and societal transformation. This exploration can help devise practical solutions to contemporary rural older adults’ care issues. In addition, analyzing older adults’ care expectations in rural areas contributes to forecasting changes in family-based older adults’ care trends, providing valuable insights for formulating comprehensive social security policies and older adults’ care services.

### 2.2 Literature review

The outflow of young labor has increasingly highlighted the issue of family-centered older adults’ care in rural areas, bringing attention to intergenerational support within family-based care. The ongoing urbanization process has significantly impacted rural family composition and population structure, exerting substantial implications for social support systems. The outflow of young labor from rural areas has placed an increasing burden on family-based older adults’ care in rural regions. Due to the late development of rural social older adult care, many older adults passively caring for themselves not only lack financial security, but also face significant uncertainty risk due to agricultural production risks, market risks, and intergenerational transfer risks (15). In light of these circumstances, two perspectives have emerged in the discussion of older adults’ care models, known as “social older adults’ care” and “family-based older adults’ care.” On the one hand, “social older adults’ care” advocates a transition from a family-centric system to a government-led social older adult care system in rural areas, advocating for the establishment of a pension system primarily based on social pension insurance (16, 17). On the other hand, “family-based older adults’ care” argues for the continuation of the family’s role in rural older adult care, citing the strengths of Chinese cultural traditions and the path dependence, cost, and performance of institutional innovation (18). Research, however, has shown that the role of the social older adults’ care insurance system is not as impactful as anticipated. Families remain the primary source of older adults’ care in rural areas. Expressly, family-based older adults’ care has seen an expansion in its manifestation, while the core roles and functions remain unchanged (19). As a result, scholars have started delving more profoundly into the current state of intergenerational support, the motivations behind it, and the relationship between intergenerational support and family-based older adults’ care amidst changing intergenerational dynamics.

Previous studies have explored intergenerational support and family-based older adults’ care within the context of three dimensions: financial provision, caregiving, and emotional solace, analyzing behavioral motivations within intergenerational interactions. Some scholars have used ordered logistic regression to analyze survey data regarding intergenerational relationships. Results show that rural residents witness an increase in emotional interaction across three generations, a decrease in financial interaction, and a trend leaning towards future generations in terms of labor support (20). The more older adult individuals contribute to grandchild care, the more their offspring tend to prefer familial older adults’ care (21). Children with better intergenerational relationships are more inclined to choose traditional home-based older adult care for their parents (22). When the New Rural Social Pension Insurance was considered as a factor, an analysis of CHARLS data indicated that it had a “crowding in” effect on financial support, a negative “crowding out” effect on temporal support, and an increase in time spent on intergenerational caregiving (23). The expansion of social security coverage has, to some extent, promoted a two-way flow of intergenerational economic resources (24).

Regarding motivations behind intergenerational support, various theories, including “reciprocity theory,” “social exchange theory,” and “kinship value theory,” emphasize the principle of reciprocity in intergenerational relationships (25). Under the influence of traditional filial piety values, intergenerational support displays characteristics of morality and ethical responsibility. However, during periods of social transformation, the logic of intergenerational support exhibited by the parental generation adheres to traditional ethical obligations, while the younger generation’s caregiving gradually loses its super-economic ethical features, leading to a significant weakening of emotional support in family-based older adults’ care (5).

Considering intergenerational changes, current rural family intergenerational relationships display the feature of “division without separation.” Intergenerational support leans towards rationalization and practices a “downward responsibility ethics” (26). In the context of older adults’ care practices, rural older adults’ care has characteristics of materialization, standardization, and clarity in exchange content (27). As the dynamic equilibrium of intergenerational relationships faces challenges in the process of social development, some scholars have started researching the factors influencing intergenerational support between the parental and offspring generations, focusing on the question of whether “raising children can provide for old age”. Huang et al. (28) analyzed the impact factors of intergenerational support from both the parental and offspring dimensions, while Nie (29) examined the influence of children’s socio-economic characteristics and intergenerational interaction on older adults’ care resource acquisition at the micro-level. The results indicate that although upward intergenerational support tends to be economically rational, intergenerational support primarily flows from children to parents. The “raising children to provide for old age” concept, to some extent, aligns with reality. Expressly, the better the intergenerational relationship, the higher the likelihood of parents receiving financial support from their children. Some studies have found that when rural older adults pay for housing and child-rearing expenses, adult children may not be able or willing to provide care for them. This could be due to cultural or structural lags among the older adults in rural areas, or because the rapid upward mobility of children leaves them without the immediate capacity to reciprocate to their parents (30, 31).

Furthermore, children’s economic conditions significantly impact the older adults’ sources of income (32), but there is no consistent conclusion regarding the impact of the number and gender of children on intergenerational support. Research has also shown that the gender of the children can influence the type of intergenerational support, with daughters tending to provide caregiving support and sons more likely to provide financial support. Sons are also more likely to receive both financial and caregiving support from their parents (33).

Intergenerational support not only affects the quality of life of the parents but also influences their expectations for older adults’ care. As social older adults’ care burdens increase and resources become scarce, the upward support provided by the younger generation significantly influences the parental generation’s older adults’ care expectations and choices. This phenomenon is particularly pronounced in single-child families (34). Research indicates that in single-child families, the correlation between the parental generation’s older adults’ care expectations and the characteristics of the offspring generation are not clear. Instead, intergenerational support and parental generation characteristics significantly influence the choices of older adults’ care subjects. The extent of the parental generation’s intergenerational support expectations is largely constrained by social norms. The alignment between older adults’ care expectations and intergenerational support significantly impacts older adults’ care perceptions and fulfillment. Support from offspring, consistent with expectations and perceived at an appropriate time, is more valued and needed by the older adults (35). Scholars, through a comparison of older adults’ attitudes towards aging and filial expectations between two distinct cultural groups, the UK and China, found that older adults with higher expectations of filial piety were more likely to adopt a positive attitude towards aging. In addition, the intergenerational care expectations and their fulfillment, which are shaped by normative constraints, are influenced by factors such as age, marital status, and health conditions (36). Moreover, instrumental and emotional support provided by offspring can be partially interchangeable when they align with parental expectations and are provided at the right time, highlighting the essential role of balance between intergenerational support and older adults’ care expectations in improving older adults’ care quality (37).

Currently, rural older adults continue to lean towards intergenerational older adults’ care as their preferred choice. Modernization has not weakened the role of children in providing older adults’ care resources and caregiving but has instead driven changes in traditional family-based older adults’ care models (38). Therefore, many studies have focused on the older adults’ care wishes and choices amidst changing intergenerational relationships. Pu and Wang (13), based on 2014 survey data on older adults’ care methods in Jilin Province, explore the influence of family structure factors on older adults’ care expectations and older adults’ care preference of residents. The authors find that the level of filial piety of children and the harmony of intergenerational relationships were critical factors. Based on the 2014 China Longitudinal Aging Social Survey (CLASS), Tao et al. (25) investigate the impact of grandchild-caring and intergenerational relationships on the older adults’ care wishes. Guo and Zhang (39), using mixed research methods, quantitatively and qualitatively analyze changes in older adults’ care expectations and their impacts under the interaction between intergenerational relationships and older adults’ care insurance.

Existing studies have mainly focused on the older adults’ care wishes and preferences amid changing intergenerational relationships, emphasizing structural changes and development trends in family-based older adults’ care. However, there is limited research that delves into the relationship between intergenerational support and older adults’ care expectations among rural residents. Existing studies have predominantly examined urban populations or are limited in applicability due to the use of localized survey data. Therefore, this paper intends to investigate the influence of intergenerational support on older adults’ care expectations in rural areas through an analysis of national-level sample data and in-depth interviews with typical cases.

## 3 Research hypotheses and methodology

Given the obvious conflicts between intergenerational care demands and supplies in rural areas, this study focuses on individuals aged 60 and above in rural regions. Intergenerational support encompasses three primary dimensions: financial, care, and emotional support. Previous research has mainly addressed the emotional dimension. This study places particular emphasis on financial and care aspects of intergenerational support and employs a combined approach involving quantitative analysis and in-depth interviews to investigate the relationship between intergenerational support and filial expectations in rural older adults’ care. To begin, we analyze national-level sample data to provide an overview of the current status of intergenerational support and rural older adults’ care expectations. Subsequently, we construct logistic models to examine the relationship between intergenerational support and older adults’ care expectations, validating the theoretical hypotheses. Finally, we integrate insights from in-depth interviews to analyze the changing trends in rural older adults’ care expectations and discuss the challenges faced in rural intergenerational care.

### 3.1 Research hypotheses

Existing literature has demonstrated that under the influence of social norms and ethical considerations, the older adults’ care expectations and filial piety expectations of the parental generation are influenced by demographic factors such as age, educational attainment, and marital status (40). Parental older adults’ care choices do not have significant correlations with children’s characteristics but are closely associated with intergenerational support and its types (5, 21). Given the heterogeneity of individuals, we posit that older adults’ care expectations may be linked to parental demographic features and intergenerational contributions. Furthermore, due to changes in family structures and the downward shift in intergenerational relationships, intergenerational exchanges between parents and children tend to become more materialistic and transactional (41). With evolving intergenerational dynamics and the decline of filial piety culture, parents no longer experience guaranteed intergenerational reciprocation (7). Expressly, there is a noticeable weakening of the family’s older adults’ care function, especially in daily caregiving and emotional solace (35, 39). As a result, the level of intergenerational support provided by children is hypothesized to be a key determinant in shaping parental older adults’ care expectations (32). Therefore, this study posits the following hypotheses:

H1: The overall level of older adults’ care expectations is associated with the degree of intergenerational support provided by parents to their offspring.

H2: Financial and care expectations for older adults’ care are positively correlated with the level of financial support provided by the offspring.

H3: The higher the parental income, the lower their reliance on financial support for older adults’ care.

H4: Educational attainment, age, and marital status have moderating effects on the impact of intergenerational support on the process of shaping older adults’ care expectations.

### 3.2 Data source

The data used in this study is derived from the 2018 CHARLS, a panel dataset. The baseline survey of this dataset covers 28 provincial, 150 county-level, and 450 village-level administrative regions across China. Employing a multi-stage stratified probability proportional to size (PPS) random sampling strategy, the CHARLS survey selects individuals aged 45 and above from each household, collecting fundamental information from three generations: the individual, their parents, and their children. The CHARLS database collects over 10,000 questionnaires annually, containing information on intergenerational financial support, care, and social interactions, which helps to deeply analyze the relationship between intergenerational support and older adults’ care expectations. After matching and partitioning the original dataset, we construct a panel dataset. In the process of data preprocessing, we exclude parents without working children to account for their inability to provide financial support. This results in a final sample of 8,127 samples.

### 3.3 Binomial logit model and variable selection

This study employs a binomial logit model to elucidate the relationship between intergenerational support and older adults’ care expectations. The choice of this model is predicated on the binary nature of the dependent variable, which represents the choice of older adults regarding their method of older adults’ care, where y=1 signifies reliance on children, and y=0 signifies not relying on children. Compared to multinomial and ordered Logit regressions, the binary Logit regression model has a distinct advantage in dealing with binary dependent variables, focusing on predicting the probability that an observation belongs to a particular category and accurately estimating the effects of the independent variables that influence this probability. The independent variables x , which influence the choices made by the older adults, include intergenerational support, gender, age, education attainment, marital status, and so on. For each dependent variable $y_{i}$ , we consider it as a realization of the random variable $Y_{i}$ . $Y_{i}$ takes the value of 1 with probability π and 0 with probability 1−π . The random variable $Y_{i}$ follows a (0–1) distribution with parameter π , and the probability mass function of $Y_{i}$ is given by:

$$\mathrm{Pr}\left[Y_{i}=y_{i}\right]=\pi_{i}^{y_{i}}{\left(1-\pi_{i}\right)}^{1-y_{i}},y_{i}=0,1$$

We define odds (the ratio of the probability of an event happening to the probability of it not happening) based on probability *π*, where $\Omega_{i}$ = $\frac{\pi_{i}}{1-\pi_{i}}$ . By taking the logarithm of odds, we assume that the probability π undergoes a logit transformation, leading to a logistic regression model:

$$\mathrm{logit}\;\Omega_{i}=\ln\Omega_{i}=\ln\frac{\pi_{i}}{1-\pi_{i}}$$

Measurement of Dependent Variables: Financial expectations (fin_exp) and care expectations (care_exp) of older adults’ care are measured based on responses to questions in the survey. The question “If you cannot work in the future, what do you think will be your main source of financial support?” measures financial expectations. The question “If you need care in your daily life, such as eating and dressing, do you have relatives or friends who can take care of you in the long term? What is their relationship to you?” measures care expectations (38). These are transformed into binary variables (0,1), where “1” represents dependence on their children, and “0” represents independence from their children.

Measurement of Core Explanatory Variables: Given that the interviewed older adults can largely take care of themselves, the independent variables include parental financial support (pa_money), grandchild-caring (pa_care), and offspring’s financial support (fin_support). The financial support from both parents and offspring in the past year is categorized into low (“1”), medium (“2”), and high (“3”) ordinal variables (42). Grandchild-caring is represented as a binary variable (0,1), where “1” indicates the presence of such care, and “0” indicates the absence of such care (23).

Measurement of Control Variables: Control variables are considered from both parental and offspring dimensions. Parental characteristics encompass gender (male), age (age), ethnicity (minority), educational attainment (education), marital status (marr), and parental income (pa_income). Offspring characteristics involve income levels (k_income). Gender, ethnicity, and parental income are categorical variables, while educational attainment, marital status, and children’s income levels are ordinal variables. Educational attainment is divided into four categories: “1” for junior high school and below, “2” for high school/technical school, “3” for junior college, and “4” for university and above. Marital status is divided into three categories: “1” for married with a spouse, “2” for divorced/widowed, and “3” for unmarried. Children’s income levels are divided into three categories: “1” for low, “2” for medium, and “3” for high.

Measurement of Moderation Variables: Based on previous literature (34), this study selects educational attainment, age, and marital status as moderation variables, generating interaction terms between educational attainment and parental financial support (interact1), age and offspring’s financial support (interact2), and marital status and offspring’s financial support (interact3). Including too many interaction terms in the model may lead to multicollinearity issues, increasing the risk of Type I errors (43), hence we selected these three interactions that have a research basis and higher statistical power.

**Figure 1:**
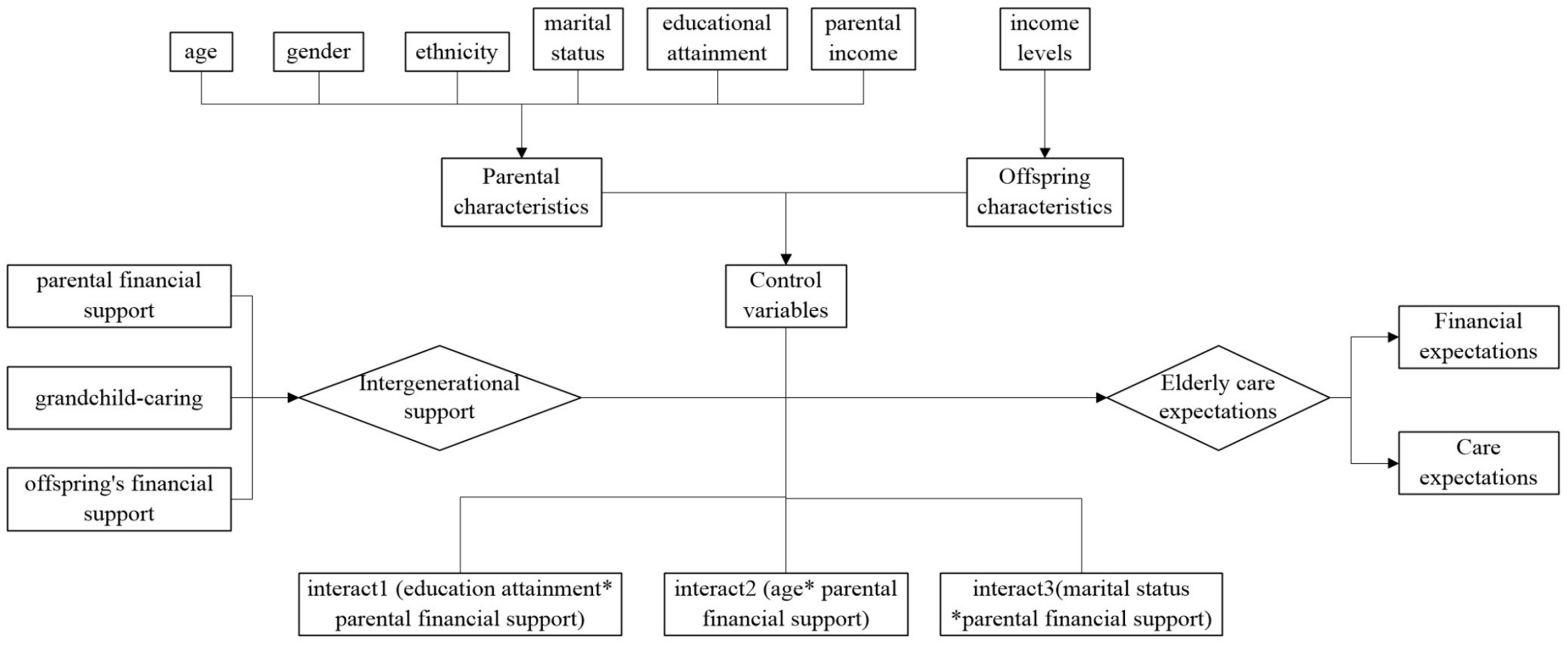
Research framework diagram.

The research framework diagram is shown in Figure 1 and the variable descriptions are shown in Table 1. All statistical analyses were performed using SPSS software and the significance level was set at *p* < 0.05 to determine statistical significance.

**Table 1:** Variable descriptions.

VarName	Obs	Mean	SD	Min	Median	Max
Age	8,127	69.07	7.107	60.00	67.00	118.00
Male	8,127	0.47	0.499	0.00	0.00	1.00
Minority	8,127	0.07	0.258	0.00	0.00	1.00
Education	8,127	1.04	0.216	1.00	1.00	4.00
Marr	8,127	1.22	0.414	1.00	1.00	3.00
Pa_income	8,127	0.14	0.344	0.00	0.00	1.00
Pa_money	8,127	1.59	0.842	1.00	1.00	3.00
Pa_care	8,127	0.01	0.096	0.00	0.00	1.00
K_income	8,127	1.93	0.885	1.00	2.00	3.00
Fin_support	8,127	1.88	0.866	1.00	2.00	3.00
Fin_exp	8,127	0.70	0.457	0.00	1.00	1.00
Care_exp	8,127	0.61	0.488	0.00	1.00	1.00

### 3.4 In-depth interviews

Research collects qualitative data through participant observation and open-ended individual case interviews to provide additional insights and explanations for the research question. The interviewees have been interviewed on the premise that they have read the informed consent form. This study has selected cases that are distinct and representative, enabling a more comprehensive understanding of the current state of older adults’ care in rural areas and uncovering older adults’ care needs and support challenges that might otherwise be overlooked. Open-ended interviews are structured to minimize barriers to understanding and expression for the older adults’ respondents and to elicit valuable information. Open-ended interviews offer a more genuine and palpable representation of older adults’ expectations regarding intergenerational care in rural areas at the micro-level. The study conducted open-ended interviews with three representative older adults residing in rural areas. Interviewee 1 is an 84-year-old widowed retired female teacher who has undergone coronary bypass surgery and is in relatively poor health. Interviewee 2 is a 59-year-old female farmer living with her son who has not purchased any form of older adults’ insurance. Interviewee 3 is a 72-year-old male farmer living alone, who receives a pension and survivor benefits.

## 4 Empirical results

### 4.1 Descriptive statistical analysis

Table 2 presents descriptive statistics of the current level of rural older adults’ financial older adults’ care expectations. It can be observed that 70.21% of the sampled rural older adults intend to rely on their children’s financial support for their future older adults’ care expenses, while 29.79% anticipate personal savings, pensions, retirement funds, and older adult care insurance or other means as their future financial sources. Overall, the majority of rural older adults still plan to rely on intergenerational financial support to finance their older adults’ care, indicating level of financial older adults’ care expectations.

**Table 2:** Financial sources of older adult care.

Financial sources of older adult care	Freq.	Percent	Cum.
Not dependent on children	2,421	29.79	29.79
Dependent on children	5,706	70.21	100
Total	8,127	100	

Table 3 provides the descriptive statistics of rural older adults’ older adults’ care expectations. The results demonstrate that 60.88% of rural older adults plan to rely on their children for caregiving, while 39.12% believe their spouses, other relatives, hired help, or nursing home staff can provide long-term care. Hence, at present, rural older adults’ care primarily depends on intergenerational caregiving by children, emphasizing the predominant role of offspring in older adults’ care choices.

**Table 3:** Care expectation of older adult care.

Care expectation of older adult care	Freq.	Percent	Cum.
Not dependent on children	3,179	39.12	39.12
Dependent on children	4,948	60.88	100
Total	8,127	100	

### 4.2 Binomial logit regression models of intergenerational support and older adults’ care expectations

The dependent variables, namely, the financial older adults’ care expectations and the care expectations of older adults’ care, are both binary categorical variables. Therefore, this study constructs binomial logit regression models based on theoretical hypotheses to explore the relationship between intergenerational support and older adults’ care expectations. The models are divided into three groups. Among them, m1 and m2 individually assess the impact of control variables on financial expectations and care expectations of older adults’ care. Subsequently, m3 and m4 introduce independent variables into the previous baseline models. Finally, m5 and m6 employ interactions between independent and control variables to measure the moderating effect of parental characteristics on intergenerational support’s impact on older adults’ care expectations.

In the results of m1, it is found that, holding other conditions constant, parental educational attainment, marital status, wage income, and offspring’s income significantly influence financial older adults’ care expectations. Meanwhile, the results from m2 indicate that parental age, gender, marital status, wage income, and offspring’s income significantly affect care expectations of older adults’ care. Therefore, older adults’ care expectations are inevitably influenced by subjective factors among the older adults. Given the heterogeneity among rural older adults, an analysis of older adults’ care expectations should not only consider the overall demands but also account for variations in individual and family socio-economic status.

Model m3 builds upon m1 by including independent variables such as parental financial support (pa_money), grandchild-caring (pa_care), and offspring financial support (fin_support). The results reveal that offspring’s financial support significantly affects financial older adults’ care expectations. The relationship between the two factors is positive correlation. According to the expectations of filial piety, rural older adults often expect their children to be their financial source in old age. Hence, the more financial assistance provided by offspring, the higher the financial expectations of the parental generation. On the other hand, Model m4 added additional independent variables, such as parental financial support (pa_money), grandchild care (pa_care), and financial support for children (fin_support), building upon the Model m2. The results show that intergenerational support does not significantly influence care expectations of older adults’ care. In conjunction with existing literature, it is known that instrumental and emotional support provided by children may, under certain circumstances, serve as substitutes for intergenerational care. This is consistent with the findings of existing research (44).

Model m5, an extension of m3, introduces interaction effects between educational attainment and parental financial support (interact1), age and offspring financial support (interact2), and marital status and offspring financial support (interact3). The regression results indicate that educational attainment negatively moderates the impact of parental financial support on financial older adults’ care expectations, while age positively moderates the influence of offspring’s financial support on financial expectations. This suggests that older adults with higher levels of education have more options for their sources of financial support in old age and, therefore, have lower expectations of financial support from their children. The case of Interviewee 1, who has a relatively high level of education, also confirmed this point, stating that her income is sufficient to support herself and explicitly mentioned “There is no need for others to provide money for my old age.” Meanwhile, with increasing age, older adults, due to physiological factors, may have higher expectations of financial support from their children.

Model m6, an extension of m4, also includes the three aforementioned moderating variables. The results show that age negatively moderates the impact of offspring’s financial support on care expectations of older adults’ care, while marital status positively moderates the influence of offspring’s financial support on care expectations. This indicates that as age increases, the association between offspring’s financial support and care expectations weakens. In fact, older adults in advanced age may face a higher likelihood of lacking care and becoming marginalized in intergenerational relationships. Adult children, due to work commitments and life pressures, may only be able to meet some of the basic financial needs of the older adults. As Interviewee 1 (84 years old) mentioned, “There is a need for someone by my side,” but the children, due to work commitments, “lack the ability to take turns caring.” Furthermore, marital status is, to some extent, related to intergenerational relationship and, consequently, moderates the relationship between offspring’s financial support and intergenerational caregiving (45).

Combining the results of the above analyses, Hypothesis 1 remains unverified, requiring further in-depth research. Hypothesis 2 is partially confirmed, indicating that financial older adults’ care expectations are influenced by intergenerational financial support. The results from m3 and m4 reveal that parental wage income is inversely related to financial older adults’ care expectations but positively related to care expectation of older adults’ care, thus confirming Hypothesis 3. The inverse relationship between financial expectation and care expectation of older adults’ care suggests a complementary relationship between offspring’s financial support and caregiving. Educational attainment and age have moderating effects on intergenerational financial support and financial older adults’ care expectations, while marital status and age moderate offspring’s financial support and care expectations of older adults’ care, confirming Hypothesis 4.

The results of binomial logit regression models are shown in Table 4.

**Table 4:** Results of binomial logit regression models.

	m1	m2	m3	m4	m5	m6
Variables	Fin_exp	Care_exp	Fin_exp	Care_exp	Fin_exp	Care_exp
Care_exp			0.87^***^		0.87^***^	
Age	−0.00	0.02^***^	−0.01^**^	0.02^***^	−0.03^***^	0.03^***^
Male	−0.10^*^	−0.20^***^	−0.07	−0.19^***^	−0.07	−0.18^***^
Minority	−0.00	−0.01	0.12	−0.02	0.13	−0.01
Education	−0.32^***^	−0.02	−0.39^***^	0.04	0.01	−0.13
Marr	0.32^***^	0.21^***^	0.31^***^	0.17^***^	0.15	−0.24
Pa_income	−0.19^***^	0.46^***^	−0.24^***^	0.50^***^	−0.24^***^	0.50^***^
Pa_money			−0.16^***^	0.07^**^	0.07	−0.03
Pa_care			0.25	0.16	0.25	0.17
K_income	0.07^**^	−0.14^***^	0.04	−0.15^***^	0.04	−0.15^***^
Fin_support			0.53^***^	**−0.03**	−0.56^*^	0.29
Interact1 (education* Pa_money)					−0.22^*^	0.09
Interact2 (age* fin_support)					0.01^***^	−0.01^**^
Interact3(marr*fin_support)					0.11	0.22^***^
Fin_exp				0.86^***^		0.87^***^
Constant	1.06^***^	−0.61^**^	0.33	−1.41^***^	1.70^***^	−1.85^***^
Observations	8,127	8,127	8,127	8,127	8,127	8,127
F						
N	8,127	8,127	8,127	8,127	8,127	8,127

* indicates *p* < 0.1, ** indicates *p* < 0.05, *** indicates *p* < 0.01. Note: Highlight notable results in bold.

### 4.3 Lack of care under adequate financial support

After revealing the statistical correlation between intergenerational support and older adults’ care expectations through quantitative analysis, this study further understands the specific contexts and motivations behind the results through in-depth interviews. The study conducted open-ended interviews with three representative older adults residing in rural areas. Interviewee 1 is an 84-year-old widowed retired female teacher who has undergone coronary bypass surgery and is in relatively poor health. Interviewee 2 is a 59-year-old female farmer living with her son who has not purchased any form of older adults’ insurance. Interviewee 3 is a 72-year-old male farmer living alone, who receives a pension and survivor benefits. All of them expressed that their retirement pensions or rural pension insurance were sufficient to cover their monthly living expenses. It is worth noting that when they referred to “pension insurance,” they were specifically referring to the rural New Rural Social Pension Insurance rather than commercial insurance. When asked if they had personally purchased pension insurance or had personal savings, interviewees two and three both responded, “Why would rural retirees need insurance? We have medical insurance in rural areas, and it costs a few hundred yuan per month.” Interviewee three added that pensions and pension insurance generally covered their regular expenses, but noted, “I can manage by myself for the most part. When it comes to medical expenses, the children help out.” This highlights that the social pension insurance system provides financial security for rural older adults to some extent. However, when confronted with substantial medical expenses, rural older adults still rely on their children for financial support.

In the backdrop of ongoing improvements in the rural social security system, the interviewees stated that they did not face financial difficulties but hoped that their children would provide care in the future. Interviewee one, as an older adults of advanced age, believed that “At present, if I do not work, there is nothing I can do, as I am physically incapable. I have back pain and discomfort in my body.” Being a widow, she lived with her son and hoped that he can spend more time accompanying and taking care of her. However, she also understood, “It’s difficult for the child as he has his own difficulties. He cannot say he’ll give up on making money for the sake of taking care of me. It’s impossible.” Throughout the interview, she emphasized the need for care from her child, but acknowledged that intense competition in society might limit the younger generation’s availability for taking care of the older adults. Due to her son’s busy work schedules, she had considered the idea of moving to a nursing home but ultimately decided against it, stating, “If you send me to a nursing home, my child will be scolded. If you have children and grandchildren, and you are sent to a nursing home, it’s a disgrace. Besides, the care in nursing homes is not good. Nursing homes are racing to the bottom. They do not respect or value the older adults. Thus, I decided to spend my old age at home.” The situation of Interviewee 1 indicates that even when the economic support provided by children is sufficient, the older adults still feel a lack of care. Economic support cannot fully substitute for emotional support and practical caregiving. These latter two needs, as higher-level requirements for the older adults, are in urgent need of being met during the process of social transformation and the improvement of rural older adults’ care services, as they directly impact the quality of life and well-being of the older adults. However, due to the constraints of filial piety on intergenerational older adult care behavior, coupled with the lack of effective oversight, poor nursing standards, and substandard living conditions in rural nursing homes, the older adults in rural areas generally have a lack of trust in social older adults’ care institutions. Even in the absence of family care, they are reluctant to choose to live in nursing homes, thus facing various uncertainties and health risks while relying on self-care.

Interviewee two adopted a relatively evasive stance on the subject of future care provided by their children, stating indirectly, “What will be will be.” Upon further probing, she indicated that she believed her son received the most significant financial and care supports from his parents, making it morally obligatory for him to care for her in her old age. This also validates the research finding mentioned earlier that “older adults’ care expectations are influenced by intergenerational economic support.” Interviewee three also commented, “We’ll see when the time comes. Can the kids avoid taking care of you?” It is clear that rural older adults still consider their children’s care as a matter of course, driven by a sense of ethical responsibility. Their actions concerning intergenerational support continue to follow the principles of reciprocal altruism. The support provided by their children in the future is viewed as a return on the investment in child-rearing within the bounds of their capabilities. However, their expectations regarding the care provided by their children remain relatively flexible, as they consider their children’s financial income, family situation, and work pressures based on emotional considerations. In light of interviewee one’s circumstances, these flexible expectations resulting from emotional tolerance might render some older adults unable to realize their care needs, potentially leading to the lack of care.

### 4.4 Intergenerational support and expectations in the “division without separation” relationship

In traditional forms of family-based older adults’ care, resources and caregiving support occur within the family, which is the primary caregiving space. However, the disintegration of extended families and the acceleration of population mobility have led to the common phenomenon of parents and children living separately, thus extending and diversifying the forms of family-based older adults’ care. This change not only reflects the direct conflict between traditional filial piety culture and modern lifestyles but also reveals the complex perspectives of the older adults regarding their own situations: they desire to maintain independence while also hoping to rely on the care provided by their children. As a result, their expectations and choices for older adults’ care become more flexible. Interviewee two, for example, indicated that her current living arrangement with her son primarily aims to take care of her grandchild, and she plans to return to her hometown as soon as the grandchild grows older. She questions, “Why would not I go back to my hometown? When the child grows up, why should I continue to live with them? What’s the point?” For her, living independently would be more comfortable if she did not need to care for her grandchild. Interviewee three expressed a similar sentiment and offered an explanation, “Because the children need their own space, and I do not want to interfere with their lives.” This indicates that the development of rural modernization has not only influenced the mindset of young people but has also led some older adults to seek individual independence and embrace the concept of personal privacy. The “division without separation” intergenerational relationship is seen as an ideal state for older adults, where caregiving by children can still occur even when they live independently.

Older adults interviewees, with the assurance of social pension insurance, primarily expect their children to provide daily care, especially when they are in poor health. Influenced by traditional ethical values, they hold higher expectations for long-term care from their sons and believe that daughters can provide temporary care if needed. If their children cannot provide daily care, the older adults’ view financial support as a compensatory form of filial piety, aligning with the findings of other scholars that there is a certain crowding-out effect between children’s financial support and caregiving. Once rural older adults receive financial support from their children, their opportunities for receiving daily caregiving from them tend to decrease.

## 5 Conclusion and discussion

This study, based on data from the 2018 CHARLS, employs binomial logit model and in-depth interview method to comprehensively analyze the impact of intergenerational support on rural older adults. The key findings of the regression models are in Table 5 and the study draws the following conclusions are as follows:

**Table 5:** Key results of binomial logit regression models.

Variables	Fin_exp		Care_exp	
	Coefficient	Significance	Coefficient	Significance
Care_exp	0.87	< 0.01		
Age	−0.03	< 0.01	0.03	< 0.01
Male	−0.07		−0.18	< 0.01
Minority	0.13		−0.01	
Education	0.01		−0.13	
Marr	0.15		−0.24	
Pa_income	−0.24	< 0.01	0.50	< 0.01
Pa_money	0.07		−0.03	
Pa_care	0.25		0.17	
K_income	0.04		−0.15	< 0.01
Fin_support	−0.56	< 0.1	0.29	
Interact1 (education* pa_money)	−0.22	< 0.1	0.09	
Interact2 (age* fin_support)	0.01	< 0.01	−0.01	< 0.05
Interact3 (marr*fin_support)	0.11		0.22	< 0.01
Fin_exp			0.87	< 0.01
Constant	1.70	< 0.01	−1.85	< 0.01
Observations	8,127		8,127	
F				
N	8,127		8,127	

First, during the period of modernization and social transformation, changes in the form of family-based older adults’ care have occurred. However, the level of intergenerational financial support significantly affects the older adults’ financial expectations of older adults’ care. Regression analysis reveals that the higher the degree of financial support from offspring, the higher the financial expectations of older adults’ care for their parents. While rural pension insurance provides a degree of financial security for older adults, they still rely on their children for significant medical expenses.

Second, upward intergenerational support does not significantly affect the overall level and degree of caregiving expectations. Regression analysis indicates that parental financial support and grandchild-caring do not significantly impact the financial or care expectations. Based on the content of the interviews, a possible reason is that parents provide financial or care assistance to their children without expecting anything in return based on ethical principles.

Third, higher parental income reduces dependency on intergenerational financial support. Some older adults with higher educational attainment or in marital relationships may have advantages in savings and social security, leading to lower dependency on their children for financial support.

Fourth, educational attainment, marital status, and age have moderating effects on offspring’s financial support and care expectations. Educational attainment negatively moderates the impact of parental financial support on financial expectations, while age positively moderates offspring’s financial support on financial expectations. Conversely, age negatively moderates offspring’s financial support on care expectations, while marital status positively moderates offspring’s financial support on care expectations. Interviews revealed that rural older adults, influenced by the concept of filial piety, continue to rely on their children as their sole support when they are unable to care for themselves, even if the expected caregiving level may not be fully achievable. Under the constraints of traditional ethical values, rural older adults often choose not to opt for social care institutions. Moreover, they have flexible care expectations, driven by emotional understanding, which might lead to the lack of care.

The above findings on older adults’ care expectations have significant implications for improving the quality of older adults’ care and enhancing the rural older adults’ support system in China. Family-based intergenerational older adults’ care, as an informal form of social support, remains a crucial component of rural older adults’ support system in China. Despite the weakening role of family-based older adults’ care, a majority of rural older adults still rely on their children. In the formulation of older adults’ care policies, greater attention should be given to supporting family-based older adults’ care and constructing a family-centric older adults’ service participation system. With the goal of meeting the needs of family development, it is essential to rationally allocate resources with coordination and assistance from both communities and institutions. With a declining birthrate and an aging population, there is a significant gap in the human resources available for family-based older adults’ care. Additionally, it is possible to draw on the relevant experiences of other Asian countries, such as Japan’s long-term care insurance system and the day care centers, short-term care centers, and home care worker dispatch centers that South Korea is progressively establishing (46), in order to meet the development needs of families and to allocate resources reasonably with the coordinated assistance of communities and institutions.

In modern society that values individual achievement and competition, due to the time constraints imposed by work and personal lives, adult children may find it challenging to promptly meet the daily needs of older adults in rural areas (47). Local governments can take the following measures: First, to promote cohabitation or proximity of children to their parents through economic compensation, tax reductions, and other incentives, with specific support measures to be detailed in real estate tax policies, land supply, housing policies, cross-province medical insurance policies, and the pricing of electricity, water, and gas, thereby reducing the overall costs for children to care for the older adults and facilitating family-based older adults’ care. Second, diverse older adults’ care models should be developed according to different family situations, such as “mutual aid older adults’ care” and “home-based older adults’ care,” to alleviate the social pressure of intergenerational support. Local governments can fully mobilize social forces by purchasing services, providing training, and financial support, encouraging social organizations and volunteers to provide spiritual comfort and daily care for the older adults without children to care for them. Third, the government should ensure the provision of basic older adults’ care services, improve the older adults’ care institution service system, and ensure that the older adults who are economically disadvantaged, disabled, or of advanced age can receive necessary living care and medical nursing services. A specific list of service items can be established, including basic living care, health monitoring, emergency rescue, etc., and differentiated services can be provided according to the different needs of the older adults.

Due to constraints such as time, energy, and research funding, this study has limitations.

(1) The sample size of individual case interviews is small, and the coverage is narrow. The quantitative analysis utilized the CHARLS dataset, which surveyed 28 provincial-level administrative regions in China, but did not cover remote areas such as Xizang and Ningxia, and had a relatively small sample of older adults over 80 years old. This may result in a certain selection bias in the sample, making it difficult to accurately reflect the older adults’ care needs and expectations of the oldest old. Future research can further conduct theoretical analysis and practical testing based on the conclusions of this study. (2) Given the cross-sectional nature of the data, we can only discuss a correlational relationship. Further research is required for a more in-depth discussion on specific mechanisms. (3) Given the uniqueness of the independent variables (such as intergenerational support, including parental and children’s financial support) and dependent variables (such as financial older adults’ care expectations and care expectations of older adults’ care) in this study, it is challenging to find suitable alternative variables. Therefore, we have not conducted robustness analysis, which remains an area for future research. Future studies can further explore these issues, while also integrating in-depth interviews to examine the impacts of different regional filial piety cultural differences on the older adults’ care expectations, and the role of community support systems in supplementing family older adult care. These investigations can contribute to a more comprehensive understanding of the complexity surrounding older adult care in rural China.

## Data Availability

Publicly available datasets were analyzed in this study. This data can be found here: http://charls.pku.edu.cn/xwdt.htm.

## References

[1] Zhang L Zeng Y Wang L Fang Y. Urban-rural differences in long-term care service status and needs among home-based elderly people in China. Int J Environ Res Public Health. (2020) 17:1701. doi: 10.3390/ijerph17051701, PMID: 32150946 PMC7084295

[2] Liu H. Trends of population aging in China and the world as a whole. Sci Res Aging. (2021) 12:1–16. Available at: https://tinyurl.com/243e6oje

[3] Han Y He Y Lyu J Yu C Bian M Lee L. Aging in China: perspectives on public health. Global Health J. (2020) 4:11–7. doi: 10.1016/j.glohj.2020.01.002

[4] Wu L Huang Z Pan Z. The spatiality and driving forces of population ageing in China. PLoS One. (2021) 16:e0243559. doi: 10.1371/journal.pone.0243559, PMID: 33428682 PMC7799793

[5] Li Q Fu Y. Intergenerational changes and rural elderly care: policy implications from an investigation in Rudong County. Jiangsu Prov Rural Econ. (2017) 8:62–9. Available at: https://tinyurl.com/23uphgk5

[6] Cao W. Comparison of the characteristics of foreign rural old-age security system and its enlightenment. World Agric. (2013) 11:115. doi: 10.13856/j.cn11-1097/s.2013.11.022

[7] Sun J. Migration of adult children and its impacts on family intergenerational relationship of the rural China. Popul J. (2010) 1:28–33. Available at: https://tinyurl.com/yk2drb7e

[8] Huang J Li F. Rural old-age security policy performance. Popul J. (2013) 1:15–21. Available at: https://tinyurl.com/yc495dz8

[9] Liu L. Improving rural elderly service system during the 14th five-year plan period: challenges and tasks. Adm Reform. (2021) 5:79–87. doi: 10.14150/j.cnki.1674-7453.2021.05.005

[10] Mu G. Reform and prospect of traditional pension plan for the aged in China. J Renmin Univ China. (2000) 5:39–44. Available at: https://tinyurl.com/2cw4dyj3

[11] Fei H. Elderly support issues in changing family structures: revisiting changes in Chinese family structure. J Peking Univ. (1983) 3:7–16. Available at: https://tinyurl.com/27xcy6ce

[12] Liang C. Collective memory, community opinion, and village welfare: the continued mechanism of rural family elderly care. Journal of. Soc Dev. (2022) 1:246. Available at: https://tinyurl.com/23v3j7ky

[13] Pu X Wang Y. Research on residents' retirement expectations and preferences under family structure changes. Popul J. (2016) 4:60–6. doi: 10.16405/j.cnki.1004-129X.2016.04.006

[14] Gui S. Rational thinking on eldercare policy to cope with aging. J East China Normal Univ. (2017) 4:163. doi: 10.16382/j.cnki.1000-5579.2017.04.011

[15] Chen F Fang C. Weakening of family elderly care function and the way out: a study on the elderly care model in undeveloped rural areas. Popul Dev. (2014) 1:99–106. Available at: https://tinyurl.com/2avrflok

[16] Yi J Lu D Deng Y. The future of social elderly care in China: from the perspective of service-oriented government. J Serv Sci Manag. (2016) 9:211–8. doi: 10.4236/jssm.2016.93025

[17] Zhang Y. Meeting the ageing challenge: China’s social care policy for the elderly. China Dev Govern. (2013):343–9. doi: 10.1142/9789814425858_0036

[18] Aboagye E Agyemang OS Tjerbo T. Elderly demand for family-based care and support: evidence from a social intervention strategy. Global J Health Sci. (2014) 6:94–104. doi: 10.5539/gjhs.v6n2p94, PMID: 24576369 PMC4825365

[19] Liu W Zhang Z. Main models of rural elderly care during the period of social transformation: social insurance or family support. J Hainan Univ. (2013) 6:103–10. doi: 10.15886/j.cnki.hnus.2013.06.019

[20] Wei H Zhong Z. Unity or alienation: intergenerational support among rural residents in the transitional period—an analysis based on micro data from national rural areas. Chin Rural Econ. (2016) 6:2–14. Available at: https://tinyurl.com/27egby2y

[21] Yang F Yang C. The effects of family structure and intergenerational exchange on individual's intention of living choice in old age. Popul J. (2016) 1:68–76. doi: 10.16405/j.cnki.1004-129X.2016.01.007

[22] Liu K Gao CZ Dai XL Li Y Liu HM. Factors influencing the choice of elderly care models from the perspective of children. Chin J Gerontol. (2021) 17:3832–5. Available at: https://tinyurl.com/28btt8he

[23] Yang R Ren Y Wang Z. New rural pension scheme, intergenerational support, and grandparenting: evidence based on the regression discontinuity design. Popul Res. (2022) 3:44–59. Available at: https://rkyj.ruc.edu.cn/CN/Y2022/V46/I3/44

[24] Wang B Zhou J. On intergenerational economic support patterns of Chinese elderly families. J Yunnan Minzu Univ. (2020) 2020:88–95. doi: 10.13727/j.cnki.53-1191/c.20200313.021

[25] Tao T Liu W Sun M. Intergenerational exchange, internalization of responsibility or altruism? The impact of grandchild-caring on the elderly’s intention for old-age support. Popul Res. (2018) 5:56–67. Available at: https://rkyj.ruc.edu.cn/CN/Y2018/V42/I5/56

[26] Fan C. Downward operation of intergenerational relationship and its impact on rural family elderly care. J Huazhong Agric Univ. (2013) 1:90–5. doi: 10.13300/j.cnki.hnwkxb.2013.01.016

[27] Li Q Yao L. Parents’kindness or children’filial piety: mode of intergenerational cooperation and its relationship adjustment in contemporary rural China. Ningxia Soc Sci. (2020) 1:106–12. Available at: https://tinyurl.com/22aogjve

[28] Huang Q Du P Chen G. The intergenerational support between adult children and older adults and its associated factors. Popul Dev. (2018) 6:128. Available at: https://tinyurl.com/233dz9nf

[29] Nie J. Can raising children provide for old age? Characteristics of children's population and economics, intergenerational relationships, and the elderly care resource acquisition of rural elderly people. J Huazhong Univ Sci Technol. (2018) 6:33–41. doi: 10.19648/j.cnki.jhustss1980.2018.06.04

[30] Qiu FX Zhan HJ Liu J Barrett PM. Downward transfer of support and care: understanding the cultural lag in rural China. Ageing Soc. (2022) 42:1422–47. doi: 10.1017/S0144686X2000152X

[31] Nie H. Excessive intergenerational support and disruption of intergenerational reciprocity: the structural dilemma of rural elderly care. Soc Sci Guangxi. (2017) 6:144–9. Available at: https://tinyurl.com/4zzwfuaf

[32] Liu L Qi L. Research on household intergenerational support and its determinants in urban China. Northwest Popul J. (2020) 4:1–14. doi: 10.15884/j.cnki.issn.1007-0672.2020.04.001

[33] Chen J. Child gender differences in intergenerational support in chinese families: the moderating role of social elderly care resources. Northwest Popul J. (2021) 6:99–112. doi: 10.15884/j.cnki.issn.1007-0672.2021.06.009

[34] Li T Fan W Song J. The household structure transition in China: 1982-2015. Demography. (2020) 57:1369–91. doi: 10.1007/s13524-020-00891-7, PMID: 32524533

[35] Feng T Ma D. Parental care subject selection by only children: a study on the impact of children's characteristics and intergenerational support. J Xi'an Jiaotong Univ. (2019) 6:76–83. doi: 10.15896/j.xjtuskxb.201906010

[36] Laidlaw K Wang DH Coelho C Power M. Attitudes to ageing and expectations for filial piety across Chinese and British cultures: a pilot exploratory evaluation. Aging Ment Health. (2010) 14:283–92. doi: 10.1080/13607860903483060, PMID: 20425647

[37] Merz EM Schulze HJ Schuengel C. Consequences of filial support for two generations: a narrative and quantitative review. J Fam Issues. (2010) 31:1530–54. doi: 10.1177/0192513X10365116

[38] Shang Q Zhao Y. Intergenerational support, social security and rural endowment mode: empirical analysis based on CHARLS. Sci Decision Making. (2022) 2:68–79. Available at: https://tinyurl.com/23vf9rp6

[39] Guo Y Zhang Y. New perspective on intergenerational relations, social pensions, and elderly care in urban China. Soc Stud. (2021) 2:229. Available at: https://tinyurl.com/2bxoyxgx

[40] Peng XZ Wang XH. The effects of family structure and personal endowment on older adults choice of aged care location. Popul J. (2021) 1:64–77. doi: 10.16405/j.cnki.1004-129X.2021.01.006

[41] Bai L Gu H. Study on the influence of intergenerational support of children on the health level of the elderly in rural areas. Modern Econ Res. (2021) 7:40–7. doi: 10.13891/j.cnki.mer.2021.07.006

[42] Tang S Yao L Li Z Yang T Liu M Gong Y. How do intergenerational economic support, emotional support and multimorbidity affect the catastrophic health expenditures of middle-aged and elderly families?-evidence from CHARLS2018. Front Public Health. (2022) 10:872974. doi: 10.3389/fpubh.2022.872974, PMID: 35462809 PMC9024169

[43] Song Y Chen Z Yan H. Can digital transformation reduce a firm's dependence on big customers? Manag Rev. (2024) 2:130–42. doi: 10.14120/j.cnki.cn11-5057/f.2024.02.010

[44] Liu X Lu B Feng Z. Intergenerational transfers and informal care for disabled elderly persons in China: evidence from CHARLS. Health Soc Care Community. (2017) 25:1364–74. doi: 10.1111/hsc.12441, PMID: 28276169

[45] Li X Zhao Z Johnson M Fang X. Intergenerational relationships and marriage in China:within‐familylongitudinal associations andbetween‐familydifferences. Pers Relat. (2023) 30:501–21. doi: 10.1111/pere.12476

[46] Yeung WJJ Lee Y. Aging in East Asia: new findings on retirement, health, and well-being. J Gerontol Series B. (2022) 77:589–91. doi: 10.1093/geronb/gbab055 33788932

[47] Chan RK Wang LR Hu K. Agentic values, generational contract, and elderly provisions in Taiwan and Hong Kong. Asian Soc Work Policy Rev. (2020) 14:197–206. doi: 10.1111/aswp.12209

